# Efficacy of Pyrethroid–Pyriproxyfen and Pyrethroid–Chlorfenapyr Long-Lasting Insecticidal Nets (LLINs) for the Control of Non-*Anopheles* Mosquitoes: Secondary Analysis from a Cluster Randomised Controlled Trial (cRCT)

**DOI:** 10.3390/insects14050417

**Published:** 2023-04-27

**Authors:** Constantin J. Adoha, Arthur Sovi, Boulais Yovogan, Bruno Akinro, Manfred Accrombessi, Edouard Dangbénon, Esdras M. Odjo, Hermann Watson Sagbohan, Casimir Dossou Kpanou, Gil G. Padonou, Louisa A. Messenger, Clément Agbangla, Corine Ngufor, Jackie Cook, Natacha Protopopoff, Martin C. Akogbéto

**Affiliations:** 1 Faculté des Sciences et Techniques, Université d’Abomey-Calavi, Abomey-Calavi 01 BP 526, Benin; yobousy@yahoo.fr (B.Y.); esdrasmahoutino@gmail.com (E.M.O.); watson.sagbohan@yahoo.com (H.W.S.); pagergil@yahoo.fr (G.G.P.);; 2 Centre de Recherche Entomologique de Cotonou, Cotonou 06 BP 2604, Benin; akinrobruno@gmail.com (B.A.); casimirkpanou@yahoo.com (C.D.K.); corine.ngufor@lshtm.ac.uk (C.N.); akogbetom@yahoo.fr (M.C.A.); 3 Faculty of Infectious and Tropical Diseases, Disease Control Department, London School of Hygiene and Tropical Medicine, London WC1E 7HT, UKnatacha.protopopoff@lshtm.ac.uk (N.P.); 4 Faculté d’Agronomie, Université de Parakou, Parakou BP 123, Benin; 5 Department of Environmental and Occupational Health, School of Public Health, University of Nevada, Las Vegas, NV 89154, USA; 6 Medical Research Council (MRC) International Statistics and Epidemiology Group, London School of Hygiene and Tropical Medicine, London WC1E 7HT, UK

**Keywords:** Interceptor G2^®^ LLIN, Royal Guard^®^ LLIN, Interceptor^®^ LLIN, Density, *Culex* spp., *Mansonia* spp., Benin

## Abstract

**Simple Summary:**

Failure to control nuisance mosquitoes may potentially affect adherence to vector control tools. In the present study, we examined the impact of two dual-active ingredient (a.i.) long lasting insecticidal nets (LLIN), namely Interceptor G2^®^ LLIN (alpha-cypermethrin-chlorfenapyr LLIN) and Royal Guard^®^ LLIN (alpha-cypermethrin-pyriproxyfen LLIN), on the density of *Culex* and *Mansonia* mosquito species as compared to Interceptor^®^ LLIN (alpha-cypermethrin-only LLIN). The study took place over two years in 60 clusters in the Zou region, Benin, with 20 clusters assigned to each of three study arms. Entomological data were collected over nine rounds up to 24 months post-net distribution. Overall, there was no evidence of a significant reduction in the density of *Culex* spp. And *Mansonia* spp. in the two dual-a.i. LLIN arms compared to the pyrethroid-only net. Both mosquito genera were found to bite more outdoors, with similar magnitudes of reduction observed in all three study arms in year 2 compared to year 1. Our findings suggest that the three types of LLINs had similar effects on the density of *Culex* spp. and *Mansonia* spp. Going forward, the development of interventions which provide control of outdoor biting mosquitoes also needs to be prioritized.

**Abstract:**

The efficacy of a vector control tool in reducing mosquito biting is crucial for its acceptability. The present study compared the vector density of *Culex* spp. And *Mansonia* spp. across clusters, which received two dual-active ingredient (a.i.) long-lasting insecticidal nets (LLINs) and a standard pyrethroid-only LLIN, and assessed the seasonality of these mosquito genera. A total of 85,723 *Culex* spp. and 144,025 *Mansonia* spp. were caught over the study period. The density of *Culex* and *Mansonia* was reduced in all three arms over the study period. There was no evidence of a significant reduction in the indoor or outdoor density of *Culex* spp. in either dual-a.i. LLIN arm as compared to the standard pyrethroid-only net arm. A similar trend was observed with *Mansonia* spp. A high density of *Culex* spp. was found both in rainy and dry seasons, while for *Mansonia* spp., this was mainly observed during the rainy season. These results suggest that the novel insecticides in the dual-a.i. LLINs did not have an additional impact on these species and that pyrethroids might still be effective on them. Further work is required to determine whether these species of mosquitoes have resistance to the insecticides tested in this trial.

## 1. Background

Until recently, pyrethroids were the only insecticides approved by the World Health Organization for use on bed nets for controlling disease-transmitting insects. The large-scale deployment of long-lasting insecticidal nets (LLINs) has been shown to reduce malaria transmission globally [1] due to the impact they have on *Anopheles* mosquito populations which spread the disease. Between 2000 and 2014, insecticide-treated nets (ITNs) contributed to an estimated 42% and 66% reduction in malaria incidence and mortality, respectively [1]. However, these gains have stalled, with no reduction recorded in global malaria cases since 2015 [2]. This may be partially due to the reduced efficacy of the nets, which may be caused by the emergence and spread of pyrethroid resistance in malaria vectors [3,4,5]. A new generation of LLINs treated with active ingredients other than pyrethroids have been developed to control pyrethroid-resistant vectors. These nets incorporate a single active ingredient (a pyrethroid insecticide) and either a synergist (piperonyl butoxide) or a second insecticide (pyriproxyfen, or chlorfenapyr), with a differing mode of action. Some of these dual-active ingredient (a.i.) LLINs have been tested in Benin, with results showing reductions of 42% and 56% in the density of *Anopheles* mosquitoes in the Royal Guard^®^ LLIN (an alpha-cypermethrin-pyriproxyfen LLIN) and Interceptor G2^®^ LLIN (an alpha-cypermethrin-chlorfenapyr LLIN) arms, respectively, compared to Interceptor^®^ LLIN (alpha-cypermethrin-only LLIN) [6]. 

Some community trials have assessed the impact of vector control tools incorporating traditional neurotoxic insecticides (pyrethroids, carbamates, and organophosphates) on populations of *Culex* spp. and *Mansonia* spp., with no great effect. For instance, no significant decline in the density of *Culex* spp. and *Mansonia* spp. was observed after the community deployment of deltamethrin-incorporated ITNs in Assam, Northeast India [7]. Moreover, a six-fold increase in the density of *Culex* spp. was observed between 2014 and 2017 in Bioko Island after both PermaNet 2.0 and Actellic 300 CS-based IRS were deployed [8]. However, to our knowledge, no Phase 3 trial has assessed the community efficacy of dual-a.i. LLINs on the density of mosquitoes other than *Anopheles*, such as *Culex* spp. and *Mansonia* spp. Indeed, these two mosquito genera are known to cause a strong biting nuisance with serious discomfort to both animals and humans [9]. They are also transmitters of several diseases [10,11,12,13], of which lymphatic filariasis is the most common in Benin, with approximately 6.6 million at-risk people [14]. This secondary analysis from a large cluster randomized controlled trial (cRCT) in Benin examined the impact of Interceptor G2^®^ LLINs and Royal Guard^®^ LLINs, compared to pyrethroid-only LLINs (Interceptor^®^) on the density of *Culex* and *Mansonia* mosquito species. While some authors showed that the peak in density of these mosquito genera was observed in the rainy season [15], others found it to occur in the dry season [16]. Given these conflicting results, we aimed at assessing the seasonality of the density of *Culex* spp. and *Mansonia* spp. in the present study.

## 2. Methods

### 2.1. Study Area

The trial was conducted in three communes in Benin: Covè (07°13′08.0400″ N, 02°20′21.8400″ E), Zagnanado (07°16′00″ N, 02°21′00″ E), and Ouinhi (07°05′00″ N, 02°29′00″ E), located in the Zou region. There are two rainy seasons, lasting from May to July and from September to November. The annual rainfall varies between 900 mm and 1250 mm. A baseline survey in 2019 showed a high density of mosquitoes with an average of 97.1 mean bites per person per night. *Culex* and *Mansonia* mosquitoes accounted for 72.2% of total bites [17]. Malaria prevalence was 42.1%, 69.1%, and 67.9% in people aged <5 years, 5–10 years, and 10–15 years, respectively [18].

**Figure 1:**
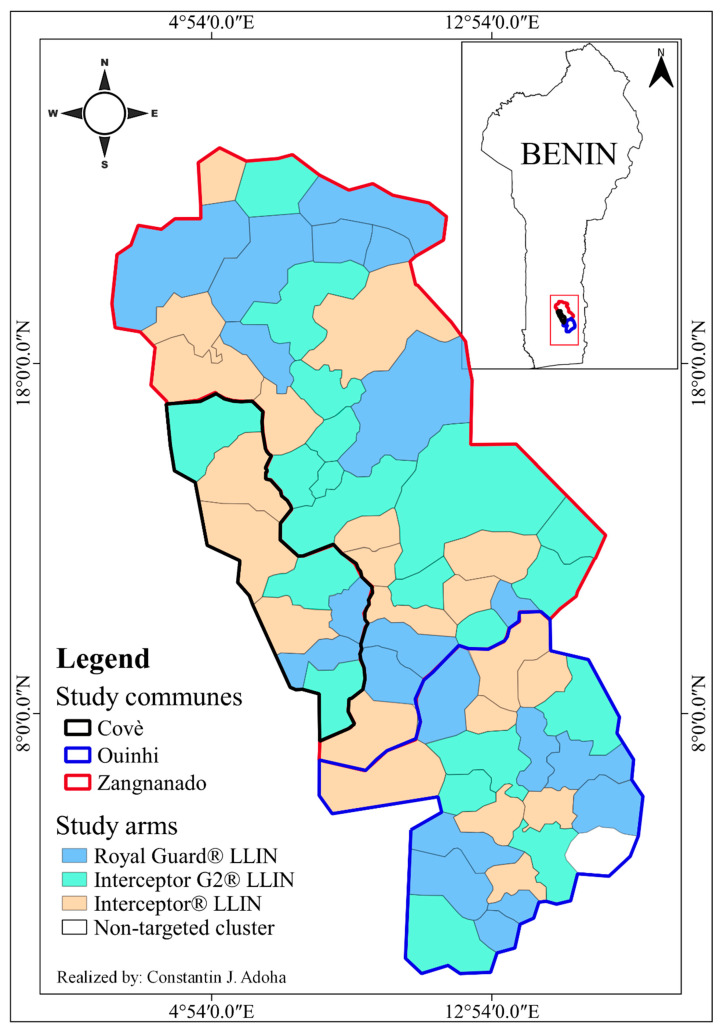
Map of the study area.

The protocol of the original trial has been described in detail elsewhere [19]. Briefly, a total of 123 villages with a population of approximately 220,000 inhabitants were divided into 60 clusters (Figure 1), each with approximately 200 households and 1200 residents. Twenty clusters were randomly allocated to each of three study arms: intervention arm 1: alpha-cypermethrin-chlorfenapyr LLIN (Interceptor G2^®^ LLIN); intervention arm 2: alpha-cypermethrin-pyriproxyfen LLIN (Royal Guard^®^ LLIN); and control arm: alpha-cypermethrin-only LLIN (Interceptor^®^ LLIN).

The total population and coverage rates of nets in the Interceptor G2^®^ LLIN arm, Royal Guard^®^ LLIN arm, and Interceptor^®^ LLIN arm were 70,989, 74,822, and 69,239 inhabitants and 97.1%, 96.4%, and 95.1%, respectively. Overall, 115,323 LLINs were distributed among the 215,050 inhabitants of the whole study area, equating to 1 LLIN for every 1.9 people.

### 2.2. Mosquito Sampling and Processing

One round of collection was performed in September–October 2019 prior to net distribution, with 8 post-intervention collections taking place between June 2020 and April 2022. In each cluster, one house was selected at random from a census list and three others at 15–20 m from the first home. In each house, two trained volunteers (one seated inside and the other outside) collected all mosquitoes landing on their legs from 19:00 to 01:00, and a second team of volunteers collected mosquitoes from 01:00 to 07:00. 

Mosquitoes were separated by genus, then morphologically identified to species level using a binocular microscope and the Gillies and Meillon [20] taxonomic key. The impact of dual-a.i. LLINs on *Anopheles* mosquitoes has been reported previously [6]; this study focused on *Culex* and *Mansonia* mosquito species. 

### 2.3. Ethical Considerations

Ethical approvals were granted by the Comité National d’Ethique pour la Recherche en Santé du Bénin (N°30/MS/DC/SGM/DRFMT/CNERS/SA, Approval n°6 of 04 March 2019) and the ethics committee of the London School of Hygiene and Tropical Medicine (16237-1). Informed written consent was sought from the heads of households as well as adult mosquito collection volunteers. Volunteers were trained to collect mosquitoes before they bite. Malaria symptoms were closely monitored, and volunteers were referred to the nearest health facility and given antimalarial drugs in case of an episode. All collectors and field supervisors were also vaccinated against yellow fever.

### 2.4. Data Management and Analysis

Entomological surveillance data were double entered into CS Pro 7.2 software and cleaned with Stata 15.0 (Stata Corp., College Station, TX, USA).

The mean number of mosquito bites per person per night was calculated for *Mansonia* spp. and *Culex* spp. at the household level. The mean density was compared between study arms using a mixed effect generalized linear model with a negative binomial distribution. Collection rounds and clusters were included in the model as random effects. The study arm was included as a fixed effect. An adjusted model, including baseline mean cluster-level mosquito density (either *Mansonia* or *Culex*) was also examined. Stata 15.0 software (Stata Corp., College Station, TX, USA) was used for the analyses.

## 3. Results

### 3.1. Mosquito Species Composition

**Figure 2:**
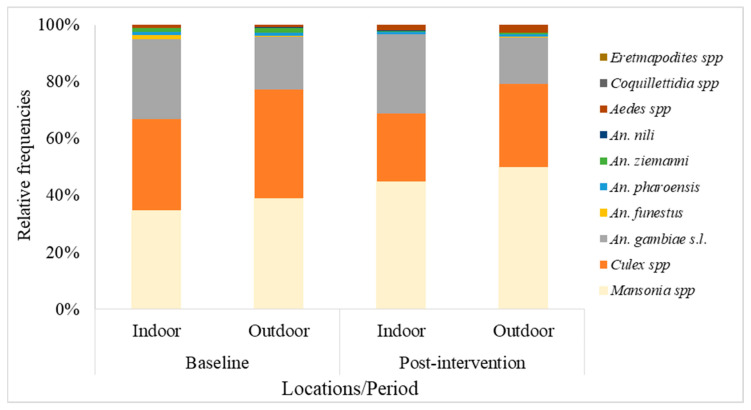
Mosquito species composition in the study area during baseline and the eight post-intervention collection rounds between September 2019 and April 2022.

In total, 331,852 mosquitoes (all species) were collected over the whole study period, with 46,613 at baseline and 285,239 over the eight collection rounds post-intervention (Figure 2). At baseline, *Mansonia* spp. and *Culex* spp. were the most abundant mosquito genera collected, with respective frequencies of 37% and 35.3%, with a greater ratio collected outdoors compared to indoors. *An. Gambiae* s.l. was the third most abundant mosquito genera collected. Other non-*Anopheles* mosquitoes collected at very low frequencies (<1%), both indoors and outdoors, included *Aedes* spp., *Coquillettidia* spp., and *Eretmapodites* spp. A similar trend was observed post-intervention (Figure 2). 

### 3.2. Density of Culex *spp.* and Mansonia *spp.* at Baseline in the Three Study Arms

Table 1 shows the densities of *Culex* spp. and *Mansonia* spp. collected per arm prior to the distribution of LLINs (baseline densities).

Overall, the indoor density of *Culex* spp. was lowest in the standard LLIN arm and highest in the pyrethroid–chlorfenapyr LLIN arm, while the outdoor density was lowest in the pyrethroid–pyriproxyfen LLIN arm and highest in the pyrethroid–chlorfenapyr LLIN arm (Table 1).

For *Mansonia* spp. at baseline, the lowest indoor density was observed in the standard LLIN arm and highest in the pyrethroid–pyriproxyfen LLIN arm. Outdoors, the density was slightly higher compared to indoors (Table 1).

### 3.3. Efficacy of Pyrethroid–Pyriproxyfen LLINs and Pyrethroid–Chlorfenapyr LLINs on Culex *spp.* Density Compared to Pyrethroid-Only LLINs

Over the whole study period, the total number of *Culex* spp. collected was 25,819 indoors and 43,450 outdoors, with the highest density caught in year 1 (Table 2). 

Overall (year 1 and 2 combined) the indoor density of *Culex* spp. in the pyrethroid–pyriproxyfen LLIN arm (15 b/p/n) was similar to the density in the standard LLIN arm (13.3 b/p/n) for both the unadjusted (DR = 0.9 (95% CI: 0.4–2.4), *p* = 0.8817) and adjusted (DR= 0.9 (95% CI: 0.4–2.0), *p* = 0.7929) models. Although the density was slightly lower in the pyrethroid–chlorfenapyr LLIN arm (11.9 bi/p/n), the reduction was not significant (DR = 0.6 (95% CI: 0.2–1.5), *p* = 0.2793 for the unadjusted model and DR = 0.4 (95% CI: 0.2–1.0), *p* = 0.0523 for the adjusted one). The same trend was observed in years 1 and 2 post-intervention. Similar observations were found outdoors (Table 2).

### 3.4. Efficacy of Pyrethroid–Pyriproxyfen LLINs, and Pyrethroid–Chlorfenapyr LLINs on the Mansonia *spp.* Density

The total number of *Mansonia* spp. collected over the collection period was higher outdoors (n = 77,036) than indoors (n = 49,759). There were more *Mansonia* mosquitoes collected in year 1 than in year 2 post-intervention, both indoors (33,102 vs. 16,657) and outdoors (50,139 vs. 26,897) (Table 3).

Overall, no significant reduction in the mean indoor density of *Mansonia* spp. was seen either in the pyrethroid–pyriproxyfen LLIN arm (28.4 b/p/n, DR= 0.5 (95% CI: 0.1–2.3), *p* = 0.3920 for the unadjusted model and DR= 0.4 (95% CI: 0.1–1.2), *p* = 0.0982 for the adjusted one) or in the pyrethroid–chlorfenapyr LLIN arm (24.4 b/p/n, DR= 0.5 (95% CI: 0.1–2.4), *p* = 0.4160 for the unadjusted model or DR= 0.5 (95% CI: 0.1–1.5), *p* = 0.2061 for the adjusted one), compared to the standard LLIN arm (25.0 b/p/n (95%: 15.4–34.5)). For each of the two post-intervention monitoring years, a similar trend was observed (Table 3).

Outdoors, no reduction in the density of *Mansonia* spp. was observed in the pyrethroid–pyriproxyfen LLIN arm (44.1 b/p/n, DR= 0.6 (95% CI: 0.1–2.6), *p* = 0.4540 for the unadjusted model and DR= 0.4 (95% CI: 0.1–1.5), *p* = 0.1825 for the adjusted one) compared to the standard LLIN arm (40.4 b/p/n (95% CI: 25.3–55.4)). In the pyrethroid–chlorfenapyr LLIN arm, there was a slight but non-significant reduction in the density of *Mansonia* spp. (35.9 b/p/n, DR= 0.6 (95% CI: 0.1–2.9), *p* = 0.5494 for the unadjusted model and DR= 0.6 (95% CI: 0.2–2.2), *p* = 0.4674 for the adjusted one) (Table 3). 

**Figure 3:**
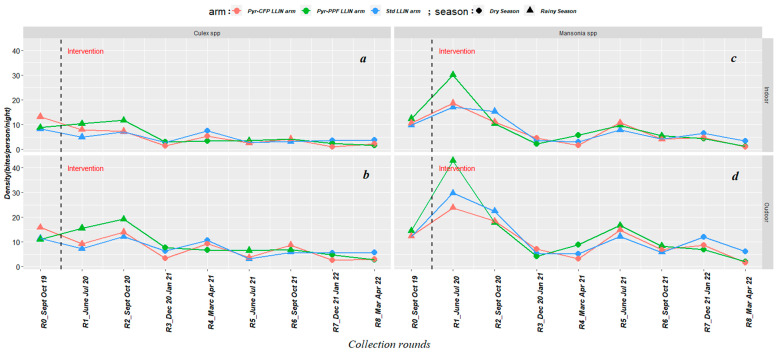
Seasonal variation of the density of *Culex* spp. (indoors (**a**) and outdoors (**b**)) and *Mansonia* spp. (indoors (**c**) and outdoors (**d**)). R: round; Std: standard; Pyr-PPF: pyrethroid–pyriproxyfen; Pyr-CFP: pyrethroid–chlorfenapyr.

For *Culex* spp., the nightly indoor density ranged between 2.8–8.3 bites/person (collection round range) in the standard LLIN arm, 1.6–11.7 b/p in the pyrethroid–pyriproxyfen LLIN arm, and 1.0–13.1 b/p in the pyrethroid–chlorfenapyr LLIN arm (Figure 3a). Outdoors, the nightly density varied between 3.1–12.2 b/p (collection round range) in the standard LLIN arm, 2.8–19.2 b/p in the pyrethroid–pyriproxyfen LLIN arm, and 2.7–15.9 in the pyrethroid–chlorfenapyr LLIN arm (Figure 3b). Overall, the indoor and outdoor density of *Culex* spp. declined over time in the three study arms, with two peaks recorded in the rainy season (September–October 2020 and 2021) and one in the dry season (March–April 2021) (Figure 3a,b). 

For *Mansonia* spp., the nightly indoor density was 3.0–17.1 b/p (collection round range) in the standard LLIN arm, 1.2–30.0 b/p in the pyrethroid–pyriproxyfen LLIN arm, and 1.0–18.7 b/p in the pyrethroid–chlorfenapyr LLIN arm (Figure 3c). Outdoors, the nightly density was 5.2–29.7 b/p, 2.0–42.8 b/p, and 1.6–23.8 b/p in the standard LLIN, pyrethroid–pyriproxyfen LLIN and pyrethroid–chlorfenapyr LLIN arms, respectively (Figure 3d). Overall, a decline in the density of *Mansonia* spp. was observed over collection rounds, with the two highest peaks occurring in the rainy season (June–July 2020 and 2021) and the lowest occurring in the dry season (December 2021–January 2022) for both indoor and outdoor collections that took place in the three study arms (Figure 3c,d). 

## 4. Discussion

This study evaluated the efficacy of a pyrethroid–pyriproxyfen LLIN and a pyrethroid–chlorfenapyr LLIN in reducing the biting frequency of *Culex* spp. and *Mansonia* spp., compared to a standard pyrethroid-only net. Overall, there was no evidence of a reduction in the density of *Culex* spp. *or Mansonia* spp. in the two dual-a.i. LLIN arms compared to the standard pyrethroid-only LLIN arm, either indoors or outdoors. The seasonal dynamics of the density of *Culex* spp. was similar in the three study arms, with a global decline observed over collection rounds and peaks in density seen in both rainy and dry seasons. The same trend was observed for *Mansonia* spp., which had a higher density. 

As previously observed at baseline by Yovogan et al. [17], *Mansonia* spp. and *Culex* spp. remained the two most abundant mosquito genera in the study area, especially outdoors. Along with *Anopheles* mosquitoes, both *Culex* and *Mansonia* are able to transmit lymphatic filariasis. A previous trial that assessed lymphatic filariasis infection in the Zou region school children aged six to seven years old, using an Alere™ Filariasis Test Strip, revealed a disease prevalence of 1.2% [19]. The high density of *Culex* spp. and *Mansonia* spp. and the endemicity of the Zou region for lymphatic filariasis [14] emphasizes the need to conduct PCR testing to assess whether the two mosquito genera play a major role in the transmission of the disease in the area, as previously shown by Lupenza et al. [21] in Tanzania and Ughasi et al. [11] in Ghana. 

Peaks in density for *Culex* spp. and *Mansonia* spp. were observed during both the rainy and dry seasons. A similar trend was previously observed by Uttah et al. [22] in Nigeria, Salako et al. [16] in Benin, and Galardo et al. [23] in Brazil. The presence of these mosquitoes all year round increases the risks of lymphatic filariasis transmission. 

In previous trials conducted in the Zambia and Papua New Guinea, ITNs showed potential for reducing lymphatic filariasis prevalence [24,25]. In the present study, while significant reductions of 42% and 56% in the density of *Anopheles* were observed through pyrethroid–pyriproxyfen LLINs and pyrethroid–chlorfenapyr LLINs, respectively, compared to standard pyrethroid-only LLINs [6], no reduction in the density of *Mansonia* spp. and *Culex* spp. was observed in either intervention arm compared to the control. Indeed, there was a decrease of similar magnitude in the density of *Mansonia* spp. in all three study arms in the second year of the trial compared to the first one. A similar trend was observed with *Culex* spp., suggesting that the three types of study mosquito net had a similar effect on each of the two mosquito genera. One possible reason for this could be that the concentration of the novel insecticides (chlorfenapyr and pyriproxyfen) incorporated into the dual-a.i. LLINs may not have been sufficient to provide an additional effect to the pyrethroid component on the density of *Culex* spp. and *Mansonia* spp. compared to the standard pyrethroid-only LLINs. Indeed, according to the WHO guidelines [26], the diagnostic concentration for an insecticide can vary from genera to genera. Ideally the dual-a.i. LLINs would be effective against *Anopheles* and other biting mosquitoes, so establishing the optimum concentration of novel insecticides (chlorfenapyr and pyriproxyfen) to apply to nets is key. 

In addition, at baseline, the two mosquito genera were found to be more exophagic than endophagic [17], which might have considerably reduced the net–vector contact, thus limiting net efficacy. A different biting behavior of *Culex* mosquitoes with similar densities both indoors and outdoors, was previously observed in the Atacora-Donga region in Benin [16]. Additionally, in Kerala State, India, *Mansonia annulifera* and *Mansonia indiana* were found to be endophagic [27]. All these results suggest that biting behavior of the *Culex* spp. and *Mansonia* spp. can vary from place to place. However, in places where these mosquitoes are exophagic or bite similarly both indoors and outdoors, outdoor control interventions, such as mosquito landing boxes [28] or attractive toxic sugar baits (ATSB) [29], could be considered. 

The decrease in density of both *Culex* spp. and *Mansonia* spp. in all three arms post-net distribution suggests also that both mosquito species might be susceptible to alpha-cypermethrin, although some studies conducted at different sites in Benin have revealed that *Culex quinquefasciatus* was resistant to pyrethroid insecticides [30,31]. This shows that the lack of insecticide resistance data in both *Culex* spp. and *Mansonia* spp. is also a limitation for the present study.

## 5. Conclusions

The concentrations of novel insecticides (chlorfenapyr and pyriproxyfen) incorporated in mosquito nets to control *Anopheles* mosquitoes might not be appropriate for *Culex* spp. and *Mansonia* spp. Additional laboratory trials are needed to determine the concentration of these insecticides applied to mosquito nets for the effective control of populations of *Culex* spp. and *Mansonia* spp. However, the reduction in density across all three arms after net distribution could mean that pyrethroids (alpha-cypermethrin) are still effective toward these species. Outdoor control interventions could also be considered for the control of these mosquito species in the areas where they are exophagic or bite similarly both indoors and outdoors.

## Figures and Tables

**Table 1:** Baseline density of *Culex* spp. and *Mansonia* spp. in the three study arms.

	*Culex* spp.			*Mansonia* spp.		
Arms	N	Person-Nights	Mean Density (95% CI)	N	Person-Nights	Mean Density (95% CI)
Indoor						
Std LLIN	1988	80	24.9 (10.4–39.3)	2346	80	29.3 (13.2–45.4)
Pyr-PPF LLIN	2105	80	26.3 (19.6–33.0)	2961	80	37.0 (21.2–52.8)
Pyr-CFP LLIN	3148	80	39.4 (15.9–62.8)	2542	80	31.8 (15.5–48.0)
Outdoor						
Std LLIN	2749	80	34.4 (21.5–47.2)	2950	80	36.9 (17.5–56.3)
Pyr-PPF LLIN	2653	80	33.2 (24.3–42.0)	3478	80	43.5 (25.1–61.9)
Pyr-CFP LLIN	3811	80	47.6 (20.7–74.6)	2953	80	36.9 (16.9–56.9)

Std: standard; Pyr-PPF: pyrethroid–pyriproxyfen; Pyr-CFP: pyrethroid–chlorfenapyr; N: number of mosquito individuals. The mean density is expressed in the number of bites/person/night (b/p/n).

**Table 2:** Impact of pyrethroid–pyriproxyfen LLINs and pyrethroid–chlorfenapyr LLINs on *Culex* spp. density.

Locations/Period	Arms	N	Person Night	Mean Density (95% CI)	DR	*p*-Value	* DR	* *p*-Value
Indoor								
Overall	Std LLIN	8541	640	13.3 (7.5–19.2)	1 (Ref)		1 (Ref)	
	Pyr-PPF LLIN	9631	640	15 (9.5–20.6)	0.9 (0.4–2.4)	0.8817	1 (0.4–2.0)	0.7929
	Pyr-CFP LLIN	7647	640	11.9 (6.4–17.5)	0.6 (0.2–1.5)	0.2793	0.4 (0.2–1.0)	0.0523
Year 1	Std LLIN	5360	320	16.8 (8.4–25.1)	1 (Ref)		1 (Ref)	
	Pyr-PPF LLIN	6840	320	21.4 (12.9–29.9)	1.0 (0.4–3.0)	0.9731	1.0 (0.4–2.5)	0.9631
	Pyr-CFP LLIN	5291	320	16.5 (8.0–25.1)	0.6 (0.2–1.7)	0.3099	0.4 (0.2–1.1)	0.0704
Year 2	Std LLIN	3181	320	9.9 (5.3–14.6)	1 (Ref)		1 (Ref)	
	Pyr-PPF LLIN	2791	320	8.7 (5.3–12.2)	0.9 (0.4–2.1)	0.766	0.8 (0.4–1.8)	0.6691
	Pyr-CFP LLIN	2356	320	7.4 (4.3–10.4)	0.6 (0.3–1.5)	0.2994	0.5 (0.2–1.0)	0.0613
Outdoor								
Overall	Std LLIN	13,627	640	21.3 (12.6–30.0)	1 (Ref)		1 (Ref)	
	Pyr-PPF LLIN	16,837	640	26.3 (17.1–35.5)	1.0 (0.4–2.5)	0.9745	1.0 (0.5–2.3)	0.9579
	Pyr-CFP LLIN	12,986	640	20.3 (11.3–29.3)	0.6 (0.3–1.6)	0.3461	0.5 (0.2–1.1)	0.0826
Year 1	Std LLIN	8726	320	27.3 (15.3–39.3)	1 (Ref)		1 (Ref)	
	Pyr-PPF LLIN	11,803	320	36.9 (22.7–51.1)	1.0 (0.4–2.8)	0.9646	1.1 (0.4–2.6)	0.8947
	Pyr-CFP LLIN	8621	320	26.9 (15.1–38.8)	0.6 (0.2–1.8)	0.4093	0.5 (0.2–1.2)	0.1341
Year 2	Std LLIN	4901	320	15.3 (8.6–22.0)	1 (Ref)		1 (Ref)	
	Pyr-PPF LLIN	5034	320	15.7 (9.2–22.3)	1.0 (0.4–2.4)	0.9279	1.0 (0.5–2.1)	0.9833
	Pyr-CFP LLIN	4365	320	13.6 (6.7–20.6)	0.6 (0.3–1.6)	0.3311	0.5 (0.2–1.1)	0.0692

*: Adjusted model; Std: standard; Pyr-PPF: pyrethroid–pyriproxyfen; Pyr-CFP: pyrethroid–chlorfenapyr; N: number of *Culex* spp. individuals; the mean density is expressed in the number of bites/person/night (b/p/n); significant threshold: *p* ≤ 0.025.

**Table 3:** Impact of pyrethroid–pyriproxyfen LLINs, and pyrethroid–chlorfenapyr LLINs on the *Mansonia* spp. density.

Locations/ Periods	Arms	N	Person-Nights	Mean	DR	*p* Value	* DR	* *p* Value
Indoor								
Overall	Std LLIN	15,979	640	25.0 (15.4–34.5)	1 (Ref)		1 (Ref)	
	Pyr-PPF LLIN	18,166	640	28.4 (16.6–40.1)	0.5 (0.1–2.3)	0.392	0.4 (0.1–1.2)	0.0982
	Pyr-CFP LLIN	15,614	640	24.4 (15.1–33.7)	0.5 (0.1–2.4)	0.416	0.5 (0.1–1.5)	0.2061
Year 1	Std LLIN	10,226	320	32.0 (19.6–44.4)	1 (Ref)		1 (Ref)	
	Pyr-PPF LLIN	12,610	320	39.4 (23.0–55.8)	0.6 (0.1–2.7)	0.5113	0.4 (0.1–1.4)	0.1628
	Pyr-CFP LLIN	10,266	320	32.1 (20.1–44.0)	0.6 (0.1–2.9)	0.5623	0.5 (0.2–1.8)	0.3207
Year 2	Std LLIN	5753	320	18.0 (10.4–25.6)	1 (Ref)		1 (Ref)	
	Pyr-PPF LLIN	5556	320	17.4 (9.1–25.6)	0.5 (0.1–2.7)	0.4007	0.3 (0.1–1.3)	0.1108
	Pyr-CFP LLIN	5348	320	16.7 (6.9–26.5)	0.4 (0.1–2.4)	0.3261	0.4 (0.1–1.5)	0.1524
Outdoor								
Overall	Std LLIN	25,832	640	40.4 (25.3–55.4)	1 (Ref)		1 (Ref)	
	Pyr-PPF LLIN	28,233	640	44.1 (26.5–61.8)	0.6 (0.1–2.6)	0.454	0.4 (0.1–1.5)	0.1825
	Pyr-CFP LLIN	22,971	640	35.9 (22.7–49.1)	0.6 (0.1–2.9)	0.5494	0.6 (0.2–2.2)	0.4674
Year 1	Std LLIN	16,247	320	50.8 (31.1–70.5)	1 (Ref)		1 (Ref)	
	Pyr-PPF LLIN	19,198	320	60.0 (35.5–84.5)	0.7 (0.1–3.0)	0.6036	0.5 (0.2–1.7)	0.2878
	Pyr-CFP LLIN	14,694	320	45.9 (29.4–62.5)	0.8 (0.2–3.4)	0.7284	0.8 (0.2–2.5)	0.6568
Year 2	Std LLIN	9585	320	30.0 (17.9–42.0)	1 (Ref)		1 (Ref)	
	Pyr-PPF LLIN	9035	320	28.2 (14.4–42.0)	0.5 (0.1–2.9)	0.4099	0.3 (0.1–1.6)	0.1674
	Pyr-CFP LLIN	8277	320	25.9 (11.3–40.5)	0.4 (0.1–2.7)	0.3752	0.4 (0.1–2.0)	0.2919

*: Adjusted model; Std: standard; Pyr-PPF: pyrethroid–pyriproxyfen; Pyr-CFP: pyrethroid–chlorfenapyr; N: number of *Mansonia* spp. individuals; the mean density is expressed in the number of bites/person/night (b/p/n), Significant threshold: *p* ≤ 0.025; seasonal dynamics of the density of *Culex* spp. and *Mansonia* spp. in the three study arms.

## Data Availability

The datasets analyzed during the present study are available on reasonable request from the corresponding authors.
